# Another debriefing course! Who benefits?

**DOI:** 10.1186/s41077-018-0087-0

**Published:** 2018-12-18

**Authors:** Kristian Krogh, Albert Chan, Nancy McNaughton

**Affiliations:** 1 Research Center for Emergency Medicine, Aarhus University Hospital, Aarhus, Denmark; 2 Department of Anaesthesia and Intensive Care, Aarhus University Hospital, Aarhus, Denmark; 3 Department of Anaesthesia and Intensive Care, Prince of Wales Hospital, Sha Tin, Hong Kong; 4 Centre for Learning, Innovation and Simulation, Michener Institute of Education at University Health Network, Toronto, Canada

**Keywords:** Debriefing practice, Feedback practice, Transfer/translate to clinical practice, Reflective practice, Continuing professional development

## Background

Not applicable

## Main text

In this editorial we argue that continuing education that is offered in brief formats (e.g. workshops), are not meeting their promise of informing practice on the ground. Using debriefing courses in simulation-based education (SBE) as an example, we discuss some concerns that result from the delivery of isolated offerings asking the question; who benefits? If our intent is to better support learning beyond our communities of health professional educators, should we think with greater purpose about how our educational efforts may be most effectively applied and translated into practice? How do we value and evaluate these continuing education activities in ways that contribute to clinical practice and better patient outcomes?

As experienced simulation educators, facilitators, and providers of these types of workshops, we strive to ensure the best possible experiences for our participants. We provide participatory exercises, demonstrations, discussions about theories, models, and principles upon which to base professional decisions and actions. We do this locally, nationally, and internationally supporting communities of practice through which we share vocabulary and knowledge about effective practices. However, for the most part, these offerings are confined to the simulation activities and do not include strategies for helping participants integrate their learning into local contexts in their own settings.

In hindsight, it seems that we may have been holding a naïve belief about our learners’ abilities to translate lessons learned in courses to their day to day practice. Argyris and Schon, two theorists with backgrounds in organizational development and professional education, have shed light on a way for us to think about this dilemma. They have identified two theories that operate within professional practice: espoused “theory of action” and “theory-in-use” which they describe as follows: “When someone is asked how he would behave under certain circumstances, the answer he usually gives is his espoused *theory of action* for that situation. This is the theory to which he gives allegiance, and which, upon request, he communicates to others. However, the theory that actually governs his actions is his *theory-in-use*” [1].

In our experience as participants, educators, and clinicians, we have noticed the “truth” of this statement. Little sustained change in practice seems to result from our various efforts. Espoused theories of action are not sustained when learners return to their own simulation and/or clinical environments. For instance, it is quite common to find educators who have learned espoused theories of debriefing in a workshop to continue to struggle with the same issues during debriefings in their respective institutions or when they attend other courses or workshops. When asked to describe the theory of action, they are often able to do so eloquently. Yet, when immersed in actual debriefings, the theory-in-use evidently is not the same.

Complicating the issue of apparent lack of transfer to practice from course participation, there is a plethora of continuing education offerings in debriefing (and other simulation techniques) from which to choose. As an example, we found that on top of all the courses being delivered internationally, the Internet is saturated with scholarly articles, research, and even YouTube presentations on different models/approaches/techniques for debriefing and accompanying rationales for why each one is the one to adopt. As of November 26, 2018, there were 6,370,000 results returned when “debriefing” was put into the Google search engine, 242,000 of these from Google Scholar. The variety of courses, research on tips, and heuristics about how to best debrief are stunning. We are not criticizing the quality or efficacy of these offerings. However, for all the effort in development and implementation of workshops and short courses and writing publications for skills building—who is benefitting? And are they benefitting as much as they could?

As educators, we all have an inherent interest in current approaches to debriefing not only for improving educational quality, but ultimately for transfer to successful patient care. Indeed, simulation educators are undoubtedly convinced of the value of simulation and its role in acquisition of technical and behavioral skills, and the expected accompanying transfer of knowledge to clinical practice. More importantly, the core value of SBE relies on the premise that it has the potential to support a change of mind and practice, enabling espoused theories of action to become theories-in-use which make actual change to practice possible.

In the current landscape of varied approaches to everything about SBE—is our focus indeed the right one—do all our efforts make the needed impact? And to what end? If we have no strategy to ensure or at least support that lessons learned can be translated into practice, what is our accountability as educators and clinicians? When things become difficult or even critical, people most often revert to their own professional practice (theories-in-use) inside their comfort zone. In other words, we need to make explicit links in our educational offerings between espoused theories of action and theories-in-use to be successful in translating educational activities to actual practice.

Perhaps, we need to take a step back and shift our focus from creating more offerings to creating more meaningful evaluations about what our offerings provide. In a thoughtful commentary about the value of SBE, Nestel et al. encourage us to look for measures of effectiveness that account for the complexity of learning that SBE provides [2]. Often transfer of learning is not linear nor is it immediate. Similar concerns were raised by McGaghie et al. about the quality of educational practice. They suggest that SBE activities need to be evaluated in light of their ability to translate learning into measurable outcomes and improved patient and population health [3]. Both perspectives support our focus on the accountability of SBE to patient care.

How do we capture meaningful change in practice from our offerings that may take years to occur? This shift in focus, from producing more educational materials to evaluating the existing offerings, does not offer the visibility to an institution or individual that delivering a course might, but may contribute far more to the field. The question becomes, as educators, should we be pressing harder for the creation of meaningful evaluations with a focus on theories-in-use rather than for the development of yet another espoused theory of action course or workshop?

In order to support deep and sustained learning, we need to examine our motivations for designing, yet another continuing education offering and tie this to the value that it may provide in practice by analyzing barriers and recalibrating our focus to the purpose of our offerings.

## Conclusions

Not applicable

## Abbreviation

SBE: Simulation-based education
